# A Biological Approach to Building Resilience and Wellness Capacity Among Police Exposed to Posttraumatic Stress Injuries: Protocol for a Randomized Controlled Trial

**DOI:** 10.2196/33492

**Published:** 2023-05-24

**Authors:** Judith Pizarro Andersen, Paula Maria Di Nota, Nazanin Alavi, Gregory Anderson, Craig Bennell, Carolyn McGregor, Rosemary Ricciardelli, Sarah Caroline Scott, Peter Shipley, Michelle Lise Vincent

**Affiliations:** 1 Department of Psychology University of Toronto Mississauga Mississauga, ON Canada; 2 Temerty Faculty of Medicine University of Toronto Toronto, ON Canada; 3 Department of Psychiatry Queen's University Kingston, ON Canada; 4 Faculty of Science Thompson Rivers University Kamloops, BC Canada; 5 Department of Psychology Carleton University Ottawa, ON Canada; 6 Faculty of Business and Information Technology Ontario Tech University Oshawa, ON Canada; 7 Faculty of Engineering and IT University of Technology Sydney Sydney Australia; 8 School of Maritime Studies Memorial University of Newfoundland St. John's, NL Canada; 9 Campus Safety Special Constable Service University of Toronto Toronto, ON Canada; 10 The Haven Mental Health Treatment Centre Port Carling, ON Canada

**Keywords:** posttraumatic stress injuries, autonomic modulation training, resilience, wellness promotion, public safety personnel, police, heart rate variability, heart rate variability biofeedback, respiratory sinus arrhythmia

## Abstract

**Background:**

Law enforcement officers are routinely exposed to hazardous, disturbing events that can impose severe stress and long-term psychological trauma. As a result, police and other public safety personnel (PSP) are at increased risk of developing posttraumatic stress injuries (PTSIs) and disruptions to the autonomic nervous system (ANS). ANS functioning can be objectively and noninvasively measured by heart rate (HR), heart rate variability (HRV), and respiratory sinus arrhythmia (RSA). Traditional interventions aimed at building resilience among PSP have not adequately addressed the physiological ANS dysregulations that lead to mental and physical health conditions, as well as burnout and fatigue following potential psychological trauma.

**Objective:**

In this study, we will investigate the efficacy of a web-based Autonomic Modulation Training (AMT) intervention on the following outcomes: (1) reducing self-reported symptoms of PTSI, (2) strengthening ANS physiological resilience and wellness capacity, and (3) exploring how sex and gender are related to baseline differences in psychological and biological PTSI symptoms and response to the AMT intervention.

**Methods:**

The study is comprised of 2 phases. Phase 1 involves the development of the web-based AMT intervention, which includes 1 session of baseline survey measures, 6 weekly sessions that integrate HRV biofeedback (HRVBF) training with meta-cognitive skill practice, and 1 session of follow-up survey measures. Phase 2 will use a cluster randomized control design to test the effectiveness of AMT on the following prepost outcomes: (1) self-report symptoms of PTSI and other wellness measures; (2) physiological indicators of health and resilience including resting HR, HRV, and RSA; and (3) the influence of sex and gender on other outcomes. Participants will be recruited for an 8-week study across Canada in rolling cohorts.

**Results:**

The study received grant funding in March 2020 and ethics approval in February 2021. Due to delays related to COVID-19, phase 1 was completed in December 2022, and phase 2 pilot testing began in February 2023. Cohorts of 10 participants in the experimental (AMT) and control (prepost assessment only) groups will continue until a total of 250 participants are tested. Data collection from all phases is expected to conclude in December 2025 but may be extended until the intended sample size is reached. Quantitative analyses of psychological and physiological data will be conducted in conjunction with expert coinvestigators.

**Conclusions:**

There is an urgent need to provide police and PSP with effective training that improves physical and psychological functioning. Given that help-seeking for PTSI is reduced among these occupational groups, AMT is a promising intervention that can be completed in the privacy of one’s home. Importantly, AMT is a novel program that uniquely addresses the underlying physiological mechanisms that support resilience and wellness promotion and is tailored to the occupational demands of PSP.

**Trial Registration:**

ClinicalTrials.gov NCT05521360; https://clinicaltrials.gov/ct2/show/NCT05521360

**International Registered Report Identifier (IRRID):**

PRR1-10.2196/33492

## Introduction

### Background

Law enforcement officers are routinely exposed to hazardous, disturbing events that can impose severe stress and long-term psychological trauma. Upward of 42% of public safety personnel (PSP) report one or more mental health symptoms [1]. Accumulated stress and posttraumatic stress injuries (PTSIs) result in chronic physical and mental health disorders, including anxiety, depression, substance abuse, and cardiovascular disease [2,3]. PTSI is also related to reduced occupational performance, absenteeism, and risky behavior, with implications for both police and public safety [4,5]. Recent empirical research and government reports have highlighted a mental health and suicide crisis among various PSP sectors in Canada [6-8]. The accumulated evidence forms an urgent call for evidence-based programs that build resilience and wellness capacity in order to prevent PTSI symptoms before they manifest as severe, chronic, diagnosable disorders in PSP.

### Biological Resilience: The Missing Piece

Existing PTSI prevention and resilience promotion interventions tailored to PSP not only show limited effectiveness but also do not consider sex and gender as central determinants of health and variables of importance [5,9,10]. Further, most traditional interventions are focused on addressing cognitive, emotional, and behavioral components without addressing the underlying neurophysiological mechanisms that erode resilience [2,11-14]. Typically, when a person encounters stress (either real or imagined), they will experience characteristic changes in the autonomic nervous system (ANS), including increased sympathetic nervous system (SNS) activation that elevates heart rate (HR) and respiration and a withdrawal of parasympathetic nervous system (PNS) activation. When stress has abated, PNS activation will lower heart and breathing rates and blood pressure, helping the body to return to reparative and restorative functioning [15].

Advances in physiology and neuroscience demonstrate that resilience is maintained by healthy ANS functioning. Disruptions in ANS functioning can be observed by objectively and noninvasively measuring 2 biological indices [13,14,16]: heart rate variability (HRV) and respiratory sinus arrhythmia (RSA). HRV is the variation in the time between successive heartbeats and depends primarily on the extrinsic regulation of HR by the balance between the SNS and PNS branches of the ANS [17,18]. If the time between heartbeats is rather constant, HRV will be low, whereas if the time varies, HRV will be higher. When a person is at rest, a high HRV signals good health because it represents a physical indicator of autonomic flexibility in responding to challenges [18,19]. In contrast, low resting HRV signals an imbalance in autonomic function, such as in clinical cases of anxiety and psychopathology, where there is an overactivation of the sympathetic branch and a weakening of parasympathetic influence over the heart [20]. RSA refers to the synchronization of HRV with breathing such that HR speeds up during inhalation (shortening the time between heartbeats) and slows during exhalation (lengthening the time between heartbeats) [21,22]. Increasing RSA builds physical wellness capacity by strengthening an individual’s long-term reserves, which is theorized to occur via multiple pathways (ie, baroreflex control, blood pressure regulation, and efficiency of pulmonary gas exchange) [21,23].

There is evidence to suggest sex can influence ANS functioning, as indicated by differences in HRV and RSA at rest [24]. These findings have direct implications for evaluating and understanding biological resilience and the effectiveness of psychophysiological interventions. Policing is known to be both intensely stressful [25] and a hypermasculinized profession [26,27], both of which have significant consequences for women’s professional experiences of stress and health. What remains to be seen is whether sociocultural gender and related occupational stressors (eg, gender discrimination and harassment) might contribute to differences in biological functioning and the effectiveness of physiologically focused wellness interventions.

### Biological Resilience Training: Heart Rate Variability Biofeedback

Research reveals a connection between respiration, arousal, and emotional control, such that how a person breathes sends signals to regions in the brainstem and forebrain that modulate arousal [28]. For example, periods of slow breathing are associated with SNS suppression and PNS stimulation, which may underlie the anxiety- and stress-reducing effects of RSA [28]. For nearly 3 decades, Lehrer and Gevirtz [21] have pioneered and refined a cardiorespiratory intervention called heart rate variability biofeedback (HRVBF). During HRVBF, a person is shown visual feedback of their beat-by-beat HR data while engaging in slow breathing with the goal of maximizing RSA. For clinically effective treatment, it is not sufficient to simply instruct a person to breathe slowly. Rather, HRVBF technology is required to identify the precise breathing rate for maximizing individual RSA and to condition this rate through training [29,30].

In collaboration with expert police practitioners as well as medical and academic experts in psychophysiological risk and resilience, the lead author developed the International Performance Resilience and Efficiency Program (iPREP), an in-person resilience program that improves occupational health and performance by addressing the unique acute and chronic stressors faced by police [31-33]. This program has been refined across 6 grant-funded multimethod studies with over 300 police participants measuring objective psychophysiological and behavioral data [31-40]. The components of the currently proposed intervention are based upon the same core scientific principles and validated intervention methods as iPREP, including mental health awareness and support [2] and psychophysiological techniques (ie, HRVBF) to promote resilience by conditioning adaptive HRV, RSA, and HR functioning [30,41,42]. Research has shown that the autonomic modulation techniques instructed during iPREP result in significant, long-term reductions in occupational errors and improvements in officers’ ability to recover quickly from acutely stressful events when completed during scenario-based, in-person training [31-33].

### Objectives

The aim of the proposed study is to develop (phase 1) and test (phase 2) if a web-based delivery of autonomic modulation training (AMT) can reduce symptoms of PTSI and build psychological wellness capacity and physiological resilience in police officers with similar effectiveness as in-person training. An additional novel scientific contribution of this protocol includes an examination of sex and gender in baseline biological and psychological presentations of PTSI among police and in response to a resilience-building intervention.

### Hypotheses

#### Self-reported PTSI Symptoms and Resilience

The following 3 hypotheses were proposed:

- H1: Symptoms of PTSI will be significantly reduced following the completion of the AMT intervention.
- H2: Functional wellness capacity and resilience will be significantly improved following the completion of the AMT intervention.
- H3: Sex and gender differences in PTSI will be observed at baseline and postintervention assessments.

Specifically, we expect that self-identified females will report more symptoms of PTSI and lower resilience at baseline, a greater reduction of PTSI symptoms, and a greater increase in resilience at follow-up than self-identified males. Gender role stress, discrimination, and harassment will be explored as moderators of PTSI symptoms at baseline and in response to the AMT intervention.

#### Objective Biological Indicators of PTSI Symptoms and Resilience

The following 4 hypotheses were proposed:

- H4: Resting HRV will significantly increase following the completion of the AMT intervention.
- H5: RSA will significantly increase following the completion of the AMT intervention.
- H6: Recovery from acute stress will significantly improve following the completion of the AMT intervention.
- H7: Based on inconsistencies in the extant literature, we will conduct exploratory analyses of any potential sex and gender differences in HRV, RSA, and acute stress recovery at baseline and postintervention assessments.

## Methods

### Ethics Approval

This protocol has been approved by the University of Toronto’s Social Sciences, Humanities, and Education Research Ethics Board (Protocol #39478).

### Design

Capitalizing on the numerous long-standing professional relationships between study coinvestigators and police populations of interest, phase 1—development of the AMT intervention—will use an integrated knowledge translation (iKT) approach to generate user-informed content [42]. To test the effectiveness of the AMT intervention (phase 2), a cluster randomized control trial with parallel assignment will be used on several cohort samples of active-duty Canadian police officers. This study has been registered with ClinicalTrials.gov (NCT05521360).

### Recruitment

We aim to test the AMT intervention on a sample of approximately 250 frontline police officers in rolling cohorts of 10-15 participants (half AMT, half waitlist control) from geographically representative police services across Canada. A power analysis was conducted using G*Power software [43] and found that a minimum total sample size of 128 participants was required (effect size f=0.25, α=.05, power=0.80, number of groups=2, number of repeated measures=2). However, we plan to oversample officers by sex and mitigate possible attrition based on the principal investigator’s previous experience with conducting longitudinal research on police officers [31-33]. A small laboratory-based prepilot sample (n=10) will test equipment reliability, accuracy, and integration of HR monitors with HRVBF and learning management system (LMS) platforms. An initial pilot cohort 1 (n=30) will then test the AMT intervention in the population of interest (ie, law enforcement officers) using a combined laboratory-remote paradigm to validate the implementation of study procedures. These participants will also be invited to participate in a poststudy focus group as per the study protocol (see iKT Focus Groups in Procedures) to optimize the intervention (ie, content and delivery) and overall study procedures to provide stakeholder- and user-informed feedback. When all user feedback has been incorporated, all subsequent participants will be tested fully remotely and in accordance with the randomized control trial procedures and group allocation.

Participants will be recruited in several ways: (1) partnering police services will provide a link to the web-based study hosted on the confidential University of Toronto (ie, not police-related) servers to members via email, announcements, and internal media postings with encouragement to participate; (2) the research team will visit police services and other related fora (eg, industry meetings and conferences) to hold recruitment and information days where eligible officers may sign up for the study; (3) our knowledge user (MLV) and expert police practitioner collaborator (PS) will facilitate recruitment of law enforcement officers through targeted media attention, networking, and professional outreach activities; (4) supporting stakeholder groups will facilitate communication and recruitment with users and law enforcement officers. Based on delays due to the COVID-19 pandemic, the order in which partner organizations are recruited and tested is subject to change based on availability and current public health guidelines.

Interested participants will be directed to a web-based consent form on a secure site that will detail the study aims, eligibility criteria, intervention protocol, allocation and procedures for experimental and control groups, risks and benefits, and other information related to their rights as a research participant (ie, confidentiality and contact information for PIs and the institutional ethics board). Participants will provide their informed consent by clicking on a link that will take them to an eligibility screening survey that will collect the following identifiable information: full name, mailing address (for equipment shipping and incentive payment), personal email address (for correspondence with the research team that is not monitored or accessible by the participants’ police agency), and demographics including sex at birth, gender, age, ethnicity, police service (to verify active duty status against publicly available directories), role, years of service as a police officer, and confirmation that they are not currently on extended medical leave. Participants deemed eligible for the study will be provided with deidentified usernames and passwords to access the preintervention assessment (see phase 2 procedures).

### Inclusion and Exclusion Criteria

Inclusion criteria included adults (aged 18 years or older) currently employed as active duty (ie, not retired or on an extended medical or disability leave) frontline law enforcement officers fluent in English. Exclusion criteria include non-Canadian law enforcement officers, police administrators, civilian employees (ie, nonsworn members), and officers that do not have regular access to a computer with internet access to complete the web-based AMT intervention. Scores on self-reported mental health screening tools completed during the preintervention sessions will be used to match the assignment of participants to experimental or control groups but will not be used to exclude participants based on established cutoff scores for high or severe PTSI symptoms.

### Procedures

#### Phase 1: AMT Development

Using an iKT approach, the multidisciplinary research team will consult with police practitioners to tailor AMT content (ie, graphics and recruitment materials) and promote participation and adherence to the AMT intervention by advising on procedural aspects of the research study (ie, feasibility, accessibility, understandability, and relevance). The AMT intervention is comprised of 2 components—HRVBF training and meta-cognitive coping skills.

#### HRVBF Training

Based on an established and publicly available protocol [30], HRVBF has shown significant efficacy in treating a wide variety of disorders, including general anxiety disorder [44-46], major depressive disorder [47-51], hypertension [52-54], asthma [29,55,56], pain [57-61], and posttraumatic stress disorder (PTSD) [62]. Recent meta-analyses revealed a large effect size for HRVBF on reducing symptoms of self-reported anxiety and stress [63] and a moderate effect for HRVBF and paced breathing across targeted physical symptoms and measures of functioning [64]. The original protocol is intended for in-person delivery, and this project will test the effectiveness of web-based HRVBF delivery among police officers. Therefore, the protocol will be formatted for web-based delivery and presented on an LMS using a commercially available HRVBF app paired with a wearable HR monitor.

#### Meta-cognitive Coping Skills

Integrated with HRVBF training, psychoeducational modules will be developed to help individuals cultivate meta-cognitive coping skills, defined as the awareness and understanding of one’s own thought processes and learning how to manage the assessment of one’s own experiences, correctly identify demands and resources, and regulate the appraisal of experiences using attentional and cognitive techniques [2]. Participants will also receive training on developing effective coping skills, including the ability to garner social support from family or friends, engage in positive relationships, and seek professional help when needed. Further, participants will receive training on emotion regulation, such as directing thoughts in the service of reducing demands or increasing resources, the ability to express emotions appropriately and keep them in perspective, and the ability to stay aware of policy and procedures under stress. These meta-cognitive coping skills are rooted in cognitive behavioral therapy principles that have been tested digitally [2] and will be developed in consultation with licensed psychiatrists and clinical coinvestigators (NA).

Research ethics approvals, data sharing agreements with partner institutions, and the procurement of equipment to record HRV and HRVBF will also be finalized during this phase of the study.

#### Phase 2: Testing the Efficacy of the AMT Intervention

##### Overview

The ention assessment, 6 weeks of AMT sessions, and a postintervention assessment. Once participants are deemed eligible for the study, they will be issued a unique deidentified username to access the web-based study, which will be hosted on secure LMS and survey platforms (eg, Qualtrics).

##### Session 1: Preintervention Assessment (1.5 Hours)

During this session participants will complete self-reported baseline measures of: (1) PTSI, including the Personal Health Questionnaire (PHQ-8) for major depressive disorder, General Anxiety Disorder Scale (GAD-7), Depression, Anxiety, and Stress Scale (DASS-42), PTSD checklist (PCL-5), Alcohol Use Disorder Identification Test (AUDIT), Perceived Stress Scale (PSS), and Personal Burnout Subscale of the Copenhagen Burnout Inventory (CBI) [65-71]; (2) resilience and well-being using the Brief Coping Orientation to Problems Experienced Inventory (Brief-COPE), Resilience Scale for Adults (RSA-33), Brief Resilience Scale (BRS), White Bear Suppression Inventory (WBSI), Perseverative Thinking Questionnaire (PTQ), Ultra Brief-Penn State Worry Questionnaire (UB-PSWQ), and Ultra Brief-Rumination Response Styles (UB-RRS) Questionnaire [72-78]; (3) functional wellness and occupational stress using the Sheehan Disability Scale (SDS), Good Health Practices Scale (GHPS), COVID-19 adapted version of the Holmes Rahe Stress Inventory (COVID-19 HRSI), World Health Organization Disability Assessment Scale (WHODAS 2.0) [79-82], operational and organizational subscales of the Police Stress Questionnaire (PSQ) [83], and Lam Employment Absence and Productivity Scale (LEAPS) [84]; and (4) sex and gender-relevant data using the Workplace Gender Discrimination Scale (WGDS) and Gender Experiences Questionnaire (GEQ) [85,86]. Global ratings of stress will be asked at the beginning of each AMT session using a sliding visual analog scale of 0 (not at all stressful) to 10 (extremely stressful) in response to 2 questions: “How stressful was this past week?” and “How stressed are you right now?.” Data will be immediately tabulated to randomly assign participants matched on demographic and outcome variables to either the experimental AMT intervention group that will continue with sessions 2-8 of the protocol or to the waitlist control group that will only be responsible for the first part of session 2 and all of session 8 (postintervention assessment).

Upon completion of session 1, participants will be asked if they want to continue in the study. This secondary “reconsent” is designed to conserve shipment of expensive study equipment (approximately USD $550 total) and retain eligible participants that demonstrate commitment to adhering to the study protocol. If participants agree to continue, they will receive a notification in their personal email confirming their mailing address and will be shipped necessary study materials, including compensation for completing session 1, a wearable HR monitor and chest band, a device equipped with the HRVBF app (an iPad), and a prepaid envelope to return all equipment at the end of the study. Once participants confirm receipt of their equipment in the mail, they will gain access to session 2.

##### Session 2: Baseline Physiological Assessment and Introduction to AMT (40-60 Minutes)

All participants will be taught via web-based video modules how to apply the wearable HR monitor and configure the HRVBF app. The wearable HR monitor will automatically record date and time-stamped physiological data upon contact with the skin via the chest band and will communicate with the HRVBF app via Bluetooth. All HRV data will be stored locally on the HRVBF app and study device, which will be mailed back to the research team at the end of the study period. In the first part of this session, both experimental and control participants will provide baseline resting HRV and RSA data that will be recorded while participants engage in a 7-minute neutral activity (eg, listening to prerecorded sounds) that does not stimulate arousal or relaxation (N Quigley, unpublished data, 2019). To assess recovery from an acute stressor, participants will perform web-based versions of the Paced Auditory Serial Addition Test (PASAT) [87] and the emotional Stroop task [88], both of which have been shown to induce significant sympathetic arousal but do not simulate traumatic material [28].

Following these baseline physiological assessments, participants in the experimental group will continue with the rest of their session (15-20 min) and begin the AMT intervention. Participants in the control group will not have any programming until session 8 (postintervention assessment).

##### AMT Intervention Sessions 3-7 (45-60 Minutes Each)

These sessions will be completed by participants in the experimental group only. The content of each AMT session will be modified from the Lehrer et al [30] HRVBF protocol and informed by practitioner, knowledge user, and stakeholder-informed design workshops to adhere to iKT principles and fit a web-based format [42]. Participants will use the HRVBF app linked via Bluetooth to their HR monitor to engage in paced breathing exercises, view their RSA progress and live HRVBF data, engage with psychoeducational content on the LMS, and complete their homework assignments (ie, paced breathing practice at least twice a day as per protocol) [30]. Participants will also view short video lectures, text, and educational content, and engage in practical exercises during each session. Participants will receive weekly emails with practice reminders and links to useful resources, including links to YouTube videos of their preferred breathing pace. Participants will learn about meta-cognitive and coping skills as they can help mitigate stress in operational, organizational, and personal contexts to foster holistic resilience and well-being.

##### Session 8: Postintervention Assessment (2 Hours)

All participants will receive an email reminder 1 week before the final session, which will be completed by both experimental and control participants. It will begin by obtaining objective physiological measures at rest, including HRV (7-minute neutral task), RSA (paced breathing), and recovery from acute stress (PASAT and Stroop tasks). Next, participants will complete the same self-reported questionnaires as in session 1 to assess changes in self-reported PTSI, resilience and well-being, occupational and operational stress exposure, and sex and gender-relevant data (see hypotheses). Participants in the control group will be asked if they would like to complete the AMT intervention. If so, they will be provided access to sessions 3 to 7 and will be asked to complete a second postintervention assessment (session 8) after completing the AMT intervention. Incentive payments will be processed as participants drop out or complete study assessments.

##### iKT Focus Groups

Following cohort 1, we will invite participants to take part in iKT Focus Groups to generate user-informed feedback on the web-based AMT intervention and identify potential sex and gender differences in the user experience. Focus groups will be led by our sex and gender expert (RR) and use a semistructured, purposive, snowball sampling design. Half-day focus groups will be held in person, with dedicated focus groups for female and male participants to provide sex-stratified samples. We will limit focus groups to 5 participants, as previous research shows groups of 4-6 ameliorate the effects of “over sharing” or domineering participants, as well as participants who may feel intimidated and become silent within larger groups [89,90]. Groups will also include officers of the same rank, a sampling stratification strategy designed to help ensure interaction with others that are not perceived as threatening and with whom an individual’s experience may also resonate [90,91]. Focus group discussions will last between 60 and 120 minutes and will be voice-recorded to preserve their accuracy during transcription.

### Outcomes

In accordance with our hypotheses, PTSI (H1) will be measured by the PHQ-8, GAD-7, DASS-42, PCL-5, AUDIT, PSS, CBI, and self-reported stress visual analog scale. Self-reported resilience and well-being (H2) will be measured by the Brief COPE, RSA-33, BRS, WBSI, PTQ, UB-PSWQ, and UB-RRS. Functional wellness capacity and occupational stress will be measured by the SDS, GHPS, COVID-19 HRSI, WHODAS 2.0, PSQ, and LEAPS. Sex will be measured by self-reported sex at birth (male, female, or other); gender is measured by self-identification as male, female, nonbinary, or other (with the option to self-report) or “prefer not to disclose.” Sex and gender-related experiences will be measured by the WGDS and GEQ (H3 and H7). Changes in resting HRV (H4) will be measured by time and frequency domain parameters based on data collected from the wearable HR monitors (eg, high- and low-frequency HRV, low- or high- frequency ratio, and the Root Mean Square of Successive Differences, [RMSSD]) as recommended in the standards of practice for HRV analysis [17]. RSA (H5) will be measured by obtaining respiratory frequency. Greater power at the respiratory frequency indicates a higher level of RSA and will be analyzed using the gold standard HRV analysis program, Kubios [92]. Recovery from acute stress (H6) will be measured following completion of the web-based PASAT and emotional Stroop Tasks. Acute stress recovery will be assessed via a time-domain measure of HRV (RMSSD) [17].

### Data Security and Analyses

Any personally identifiable information provided in the eligibility questionnaire will be downloaded and immediately deleted from web-based platforms, stored offline in encrypted files on password-protected computers at the University of Toronto accessible only by members of the primary research team approved by the lead principal investigator (JPA), and will not be shared with collaborators or participating police organizations. Data collected on the LMS and secure survey platforms (ie, pre- and postintervention assessments and responses to intervention exercises) will be matched to a deidentified user ID. Physiological data gathered by the wearable HR monitor and HRVBF app will be stored locally and will not be transferred to publicly accessible third-party cloud-based servers. Rather, participants will mail the equipment back to the research team, which will match the deidentified data offline. The key code linking participants’ personal information and deidentified user IDs will only be stored on secure servers at the University of Toronto and will be destroyed once the participant completes or withdraws from the study, has received their compensation, and all deidentified data files have been matched.

Physiological data will be transferred to a 2-time Canada Research Chair in Health Informatics and Big Data expert coinvestigator (CM) via a secure virtual private network (VPN) tunnel and housed on a private cloud-based Big Data analytics platform located within the *Compute Ontario Advanced Research Computing* equipment at the Centre for Advanced Computing (Queen’s University). CM will design the data management, cleaning, and analysis protocols necessary to construct the big data pipeline to process the large amount of continuous data, including matching the time stamps on the physiological data to significant event markers in the AMT intervention (eg, questionnaires, PASAT, and emotional Stroop tasks) and on the HRVBF app (eg, paced breathing exercise). Preprocessed data will then be sent back to the primary research team (JPA, PMD, and SCS) via a secure VPN tunnel and housed on the secure internal University of Toronto server (RES-NAS) for the statistical analyses described below using bespoke syntax and code on various software platforms (eg, Kubios, MatLab, and SPSS). Data from iKT focus groups will be stored in RR’s secure data web-based Al Fresco space and hard copies in her triple-locking filing cabinet in her office at the Memorial University of Newfoundland.

To test the effectiveness of the AMT intervention on reducing PTSI symptoms and improving resilience, we will conduct prepost analyses of the self-reported PTSI (H1) and resilience measures (H2) using separate repeated measures analyses of covariance (ANCOVAs) accounting for demographic covariates for normally distributed data, and ranked repeated measures ANCOVAs for nonnormally distributed data [93]. Variables including sex, gender (H3), age, years of service, and geographic service region (eg, urban or rural; East, Central, or West) will be included as covariates.

To test the effectiveness of the AMT intervention on improving biological indicators of resilience, prepost analyses of resting HRV parameters (H4), RSA (H5), and recovery from acute stress (H6) will be separately assessed using repeated measures ANCOVAs with sex, gender (H7), age, years of service, and geographic service region as covariates. To determine which factors significantly predict greater changes in biological outcome measures, hierarchical linear regression analyses will be undertaken with self-reported measures and demographic variables entered as predictors in the models. We will also compare weekly changes in HRV and RSA as measured during AMT module exercises with repeated measures ANCOVAs. A main effects analysis of the “time” variable will explore the potential incremental benefit of AMT training and identify at what point during the 6-week intervention significant biological changes occur. Computational approaches to analyzing and visualizing biological indices of resilience will be led by CM, including integration of cardiac (eg, HRV) and respiratory (eg, RSA) rhythms to elucidate patterns of desynchronization before, during, and following AMT.

To explore sex and gender-based contributions to self-report (H3) and objective indices (H7) of PTSI and resilience, the above analyses will include sex and gender as covariates or predictor variables with sex-stratified samples. These findings will clarify any sex-related differences in baseline and postintervention rates of PTSI and resilience among law enforcement officers and whether there are any sex-related differences in the magnitude of treatment effects (H3). Moderated regression analyses will test whether the AMT intervention affects the relationship between sex and PTSI symptoms and resilience. Multiple comparisons will be adjusted with Bonferroni corrections, where applicable.

Qualitative analyses of the transcribed data obtained from the iKT focus groups will be led by the sex and gender coinvestigator (RR) with the assistance of graduate students and postdoctoral fellows, with the aim of identifying sex- and gender-based considerations for developing effective occupational health interventions tailored to police officers and other PSP groups. Qualitative data analysis will use an inductive, comparative approach without initially arriving at any definitive, substantive, or theoretical conclusions about what the data reflect interpretatively with a lens on sociology and criminology [94]. Themes will emerge through the dynamic interaction of participants. Coding will be performed using NVivo qualitative analysis software (Lumivero), allowing for comparison across sessions [89] and the emergence of prominent themes through the tracking of coding “nodes” both across and within groups. Regular research team meetings will ensure that thematic development emerges in a consistent and reliable manner and help to ensure the hermeneutically attuned validity of the data [90].

## Results

The study received notice of funding acceptance in March 2020. In February 2021, ethics approval was obtained from the University of Toronto’s Social Sciences, Humanities, and Education Research Ethics Board (Protocol #39478) and was renewed in February 2023. Phase 1 of the study (AMT Development) was completed in December 2022. Participant recruitment for phase 2 began with the pilot cohort in February 2023 via advertisements through partnering stakeholders (see Recruitment section). As of April 2023, 7 pilot participants were actively registered for the AMT intervention. Knowledge dissemination for phase 1 was completed in March 2023 at an annual meeting of the public safety wellness team grant holders and is planned for 1 scientific (May 2023) and 1 practitioner (October 2023) conference. iKT focus groups will take place following the completion of each sufficient cohort (see the iKT Focus Groups section above).

## Discussion

Based on the extant and precedent literature, we predict that the stated hypotheses will be confirmed following the execution of the proposed protocol. Specifically, we anticipate significant reductions in PTSI symptoms and improvements in physiological indicators of health and resilience following the completion of the 6-week web-based AMT intervention. We also anticipate sex- and gender-based differences in pre- and postintervention outcomes, such that self-identified females will report higher PTSI symptoms and lower resilience at baseline and a greater reduction of PTSI symptoms and greater increase in resilience at follow-up than self-identified males. The direction and magnitude of sex- and gender-based differences in physiological outcomes are not currently specified, given the novelty of these analyses. Insights derived from iKT focus groups will supplement the quantitative sex- and gender-based analyses (H3 and H7).

AMT fills an existing gap in the applied research literature supporting evidence-informed prevention and intervention programs for PSP exposed to PTSI, such that AMT aims to measure and strengthen objective biological resilience [12]. Specifically, we are developing a tool that integrates wearable technology, real-time app-based visualized biofeedback tailored to an individual’s biological rhythms, and confidential web-based resources that meet the needs of accessibility and availability expressed by PSP organizations and governmental agencies [16]. Furthermore, our project addresses population-specific needs, including stigma reduction and gendered perspectives on occupational and organizational stress from female officers. By delivering our program on an LMS platform that can be accessed remotely in the privacy of one’s own home, we are providing officers with a secure and accessible wellness promotion intervention and resources that they might not otherwise seek out. This project will also contribute substantial new knowledge to the scarce literature about how sex and gender are related to biological indicators of PTSI and resilience (ie, HRV and RSA). Specifically, analyses will reveal the directionality and strength of interactions between sex and gender and physiological resilience outcomes (eg, what was thought to be sex-based differences in HRV may be related to gender norms and gender stress; see [95]). Our study provides a well-powered design to sufficiently address the persistent knowledge gap in this area.

AMT techniques have previously garnered high “buy-in” from participating police officers because the intervention provides tangible, real-time, biological evidence of occupational and health improvement from the very first HRVBF session [31-34]. Buy-in is further promoted by potentially relieving some symptoms early on without having to wait until the end of the intervention. Further, AMT directs focus on more “physical” rather than “psychological” symptoms to reduce the association of PTSI with potential weakness or occupational inadequacy among police, which has been previously noted [33,96].

Pending significant results, AMT can be delivered alone or in tandem with other psychotherapeutic treatments, such as cognitive behavioral therapy, to provide PSP services with an attractive suite of resilience and wellness capacity interventions that reduce PTSI symptoms and save operating costs (eg, maintenance fees for multiple participant licenses) [97]. The intervention, if found to be effective, can be implemented on a larger scale with affordable, commercially available wearable technology (eg, FitBit and Apple iWatch) and apps (eg, HRV Trace and HRV Train) [98].

Rapid and direct knowledge dissemination to various stakeholders (eg, police organizations, unions, and professional and nonprofit first responder organizations such as The Haven, Badge of Life Canada, Ontario Women in Law Enforcement, and Wounded Warrior) will be facilitated by knowledge users’ (MLV and PS) capacities as retired PSP, board members, and English-French bilingual contributors to police periodicals including “The Blue Line.” In addition to this protocol, several research articles will be prepared based on the planned objectives and analyses identified above. High-impact peer-reviewed journals in relevant fields (eg, Psychophysiology, International Journal of Environmental Research and Public Health, and Policing) that provide open access options will be prioritized. Research findings on the pilot (n=30) and complete data sets (n=250) will be disseminated at scientific (Association for Applied Psychophysiology and Biofeedback, Society for Psychophysiological Research) and applied practitioner conferences (Law Enforcement and Public Health, Canadian Institute for Military and Veteran Health Research, Canadian Association of Chiefs of Police, Canadian Association of Police Educators) to distribute new knowledge generated to the widest audience possible.

## Appendix

Multimedia Appendix 1 Peer review from the Canadian Institutes of Health Research (CIHR).

## Abbreviations

AMT: autonomic modulation training
ANCOVA: analysis of covariance
ANS: autonomic nervous system
AUDIT: Alcohol Use Disorder Identification Test
BRS: Brief Resilience Scale
CBI: Copenhagen Burnout Inventory
COVID-19 HRSI: COVID-19 adapted version of the Holmes Rahe Stress Inventory
DASS-42: Depression and Anxiety Stress Scale-42 Item
GAD-7: General Anxiety Disorder Scale-7 Item
GEQ: Gender Experiences Questionnaire
GHPS: Good Health Practices Scale
HR: heart rate
HRV: heart rate variability
HRVBF: heart rate variability biofeedback
iKT: integrated knowledge translation
iPREP: International Performance Resilience and Efficiency Program
LEAPS: Lam Employment Absence and Productivity Scale
LMS: learning management system
PASAT: Paced Auditory Serial Addition Test
PCL-5: PTSD Checklist (DSM-5)
PHQ-8: Personal Health Questionnaire-8 Item
PNS: parasympathetic nervous system
PSP: public safety personnel
PSQ: Police Stress Questionnaire
PSS: Perceived Stress Scale
PTQ: Perseverative Thinking Questionnaire
PTSD: posttraumatic stress disorder
PTSI: posttraumatic stress injury
RSA: respiratory sinus arrhythmia
RSA-33: Resilience Scale for Adults-33 Item
SDS: Sheehan Disability Scale
SNS: sympathetic nervous system
UB-PSWQ: Ultra Brief-Penn State Worry Questionnaire
UB-RRS: Ultra Brief-Rumination Response Styles
WBSI: White Bear Suppression Inventory
WGDS: Workplace Gender Discrimination Scale
WHODAS 2.0: World Health Organization Disability Assessment Scale 2.0

## Data Availability

To protect the privacy and confidentiality of participants, which are comprised of active-duty Canadian police officers, the data sets generated or analyzed during this study are not publicly available, but deidentified or aggregated data may be available from the principal investigator (JPA) on reasonable request.
