# Supplementary material for: A Biological Approach to Building Resilience and Wellness Capacity Among Police Exposed to Posttraumatic Stress Injuries: Protocol for a Randomized Controlled Trial
Source: JMIR Res Protoc. 2023 May 24;12:e33492. doi: 10.2196/33492 (PMC10248782; doi:10.2196/33492)
Supplement: Multimedia Appendix 1 [file resprot_v12i1e33492_app1.pdf]

Canadian Institutes of Health Research / Instituts de recherche en santé du Canada

Notice of Decision / Avis de décision

Application Number/Numéro de la demande: 440144

Committee Code/Code du comité: MWP

Applicants/Candidats: Dr. Judith Andersen

With/Avec: Dr. N. Alavi, Dr. G. Anderson, Prof. C. Mcgregor, Dr. R. Ricciardelli, Ms. M. Vincent

Institution paid/ Établissement payé: University of Toronto

Title/Titre: Autonomic Modulation Training: A Biological Approach to Building Resilience and Wellness Capacity Among Police Exposed to Post-Traumatic Stress Injuries (PTSI)

Primary Inst./ Inst. principal: Neurosciences, Mental Health and Addiction / Neurosciences, santé mentale et toxicomanies

Other Related Inst./ Autres inst. connexes:

Competition Outcome/Résultats du concours: Team Grant: Mental Wellness in Public Safety Team Grants - Police / Subv. d'équipe : Bien-être mental du personnel de la sécurité publ. - Policiers

November/Novembre 05, 2019

Number in competition/Nbre de demandes dans le concours: 3

Number approved/Nbre de demandes approuvées: 1

Decision on your application/ Décision sur votre demande: Approved / Approuvée

Average annual amount/ Montant annuel moyen: $328,667

Equipment amount/ Montant pour les appareils: $4,000

Term/Durée: **3 yrs/ans 0 months/mois**

Peer Review Committee Recommendation, for your information and use/ Recommandation du comité d'examen par les pairs, pour fins d'information et d'utilisation:

Committee/Comité: Team Grant: Mental Wellness in Public Safety Team Grants – Full Application / Sub. d'équipe: Sub. d'équipe sur le bien-être mental du personnel de la sécurité publique

Application rank within the competition/ Rang de la demande dans ce concours: 1

Percent Rank Within the Competition/ Rang en pourcentage au sein du concours: 33.33%

Rating/ Cote: 3.83

Recommended average annual amount/ Montant annuel moyen recommandé: $328,667

*** Applications receiving a score of less than 3.5 on any evaluation criteria will not be considered for Funding. / Les demandes qui ont reçu une note inférieure à 3.5 pour n'importe quel des critères d'évaluation ne sont pas admissibles.

This document is for information only. Official payment is stated on the CIHR Authorization for Funding.
Document à titre d'information seulement. Le paiement officiel est indiqué sur l'autorisation de financement des IRSC.

Canadian Institutes of Health Research
160 Elgin Street, 9th Floor
Address Locator 4809A
Ottawa, Ontario K1A 0W9

Instituts de recherche en santé du Canada
160, rue Elgin, 9e étage
Indice de l'adresse 4809A
Ottawa (Ontario) K1A 0W9

Institute of Aging
Institute of Cancer Research
Institute of Circulatory and Respiratory Health
Institute of Gender and Health
Institute of Genetics
Institute of Health Services and Policy Research
Institute of Human Development and Child and Youth Health
Institute of Indigenous Peoples' Health
Institute of Infection and Immunity
Institute of Musculoskeletal Health and Arthritis
Institute of Neurosciences, Mental Health and Addiction
Institute of Nutrition, Metabolism and Diabetes
Institute of Population and Public Health

Institut du vieillissement
Institut du cancer
Institut de la santé circulatoire et respiratoire
Institut de la santé des femmes et des hommes
Institut de génétique
Institut des services et des politiques de la santé
Institut du développement et de la santé des enfants et des adolescents
Institut de la santé des Autochtones
Institut des maladies infectieuses et immunitaires
Institut de l'appareil locomoteur et de l'arthrite
Institut des neurosciences, de la santé mentale et des toxicomanies
Institut de la nutrition, du métabolisme et du diabète
Institut de la santé publique et des populations

March 31, 2020

Dr. Judith Andersen
3359 Mississauga Rd, North
University of Toronto, Mississauga
William Davis Building RM 2037B
Mississauga, Ontario
L5L 1C6

Dear Dr. Andersen:

On behalf of the Canadian Institutes of Health Research (CIHR) we are pleased to inform you that your recent application submitted to the Team Grant: Mental Wellness in Public Safety Team Grants – Funding Pool Police, entitled "Autonomic Modulation Training: A Biological Approach to Building Resilience and Wellness Capacity Among Police Exposed to Post-Traumatic Stress Injuries (PTSI)." has been approved for funding.

Documentation pertaining to the review of your application can be found through ResearchNet. Please note that your Authorization for Funding will follow electronically.

As CIHR does not notify co-applicants of the decision, we ask that you inform those individuals involved, along with their research institutions (if different from your own), of the outcome of this application.

Should you require additional information, please contact the CIHR Contact Centre at support-soutien@cihr-irsc.gc.ca. Please do not contact the officers or members of the peer review committee.

Congratulations on your success in this competition.

Sincerely,

Nathalie Gendron

Nathalie Gendron, Ph.D.
Manager, Program Design and Delivery
Research Program Portfolio

Canada

CIHR IRSC
Discoveries for life / Découvertes pour la vie

Review Type/Type d'évaluation: Committee Member 1/Membre de comité 1
Name of Applicant/Nom du chercheur: Andersen, Judith
Application No./Numéro de demande: 433650
Agency/Agence: CIHR/IRSC
Competition/Concours: 2019-11-05 Team Grant: Mental Wellness in Public Safety Team Grants/Subvention d'équipe : Subventions d’équipe sur le bien-être mental du personnel de la sécurité publique
Committee/Comité: Team Grant: Mental Wellness in Public Safety Team Grants – Full Application/Sub. d’équipe: Sub. d’équipe sur le bien-être mental du personnel de la sécurité publique
Title/Titre: Autonomic Modulation Training: A Biological Approach to Building Resilience and Wellness Capacity Among Police Exposed to Post-Traumatic Stress Injuries (PTSI)

Assessment/Évaluation:

This proposal aims to evaluate the efficacy of a web-based autonomic modulation training intervention (i.e., real-time biological heart-rate-variability biofeedback) to mitigate mental health symptoms of PTSI in police officers. The researchers contend that traditional interventions typically focus on cognitive, emotional, and behavioral aspects of PTGI and do not address neurological/physiological mechanisms such as autonomic nervous system regulation. Overall, the application is well-written and the aims are well-developed and justified.

Below, this reviewer provides comments according to the evaluation criteria for this review.

1. Impact of the Research

The proposed research has the potential to provide new knowledge regarding the potential efficacy of a within-subjects autonomic modulation training intervention in police officers with PTGI. This approach is unique in its focus on “bottom up” autonomic dysregulation of PTGI instead of “top down” cognitive, emotional, and behavioral mechanisms that typify the focus of contemporary CBT approaches. The proposed aims are systematic and integrative, and follow established methods in the field.

The proposal integrates sex and gender, and will employ a gender-sensitive approach at all phases of the study. The research team provides a detailed background on sex and gender considerations related to their core measures of HRV and RSA, which provides a clear demonstration of their grasp of the importance of sex as a potential effect moderator.

The potential net result of the proposed research is a scalable intervention that targets autonomic nervous system dysregulation in police officers and related occupational groups with PTSI. Given issues of mental health stigma in this population, the proposed research, with its focus on a biological mechanism underlying PTSI, has the potential to be taken up more readily than interventions focused on “softer” mechanisms, such as emotions, cognition, and behaviors. Further, this intervention can be deployed as a primary or adjunctive treatment by mental health clinicians working with police officers and other occupational groups affected by PTSI.

The research team has established relationships with several police forces across Canada, which is a notable strength of the proposed study. If found to be effective, their proposed intervention has the potential to be disseminated quite broadly, thus helping to mitigating symptoms of PTSI and bolster resilience and wellness in the broader population of police officers in Canada.

2. Approach

**Review Type/Type d'évaluation:**	Committee Member 1/Membre de comité 1
**Name of Applicant/Nom du chercheur:**	Andersen, Judith
**Application No./Numéro de demande:**	433650
**Agency/Agence:**	CIHR/IRSC
**Competition/Concours:**	2019-11-05 Team Grant: Mental Wellness in Public Safety Team Grants/Subvention d'équipe : Subventions d’équipe sur le bien-être mental du personnel de la sécurité publique
**Committee/Comité:**	Team Grant: Mental Wellness in Public Safety Team Grants – Full Application/Sub. d’équipe: Sub. d’équipe sur le bien-être mental du personnel de la sécurité publique
**Title/Titre:**	Autonomic Modulation Training: A Biological Approach to Building Resilience and Wellness Capacity Among Police Exposed to Post-Traumatic Stress Injuries (PTSI)

Assessment/Évaluation:

The proposed design of the study is appropriate and will employ state-of-the-art methods to evaluate study aims. The literature review is complete and up-to-date, and demonstrates the team’s in-depth understanding of the broader scientific literature on the role of autonomic nervous system dysregulation in PTGI, and biofeedback intervention approaches that may help mitigate this dysregulation. The roles and responsibilities of the Sex and Gender Champion identified adhere to the best practices outlined by the Institute of Gender and Health. The research team clearly articulated the limitations of their proposed study, as well as approaches to address them, most notably to address issues related to mental health stigma, which is among the most salient challenges to working with mental health issues in correctional workers.

The most notable limitation of the proposed study is the lack of an active control (or even a TAU or wait-list control) condition, which will ultimately make it impossible to determine the relative efficacy of the proposed intervention. Given the large-scale nature of the proposed study, the research team may consider potentially including such a condition, which would provide a more rigorous test of the efficacy of this intervention approach in treating PTSI.

Another potential limitation is the extent to which the police officers who will be recruited into the study are representative of the broader population of police officers. Given issues of mental health stigma, do the researchers anticipate recruiting a healthier/sicker sample? How might this impact generalizability of results? What measures are in place to ensure broader generalizability of study results? Related to this point, do the inclusion/exclusion criteria allow for recruitment of a sample that is representative of the target population?

Given the potential for police officers to be exposed to traumatic and stressful events as part of their work, what procedures are in place to assess these exposures over the course of the study, most notably between pre- and post-assessments, which may influence autonomic measures and mental health symptoms?

Has the research team considered the potential effects of sleep, recreational drug use, and medical conditions (e.g., cardiovascular disease) on study outcomes?

Minor comment: How will participation in other treatment(s), most notably those affecting autonomic nervous system function (e.g., beta blockers), be monitored and managed in the research strategy/data analysis?

It is unclear why analyses will be stratified by sex/gender. Why not incorporate sex/gender as a potential main effect and moderating variable?

The research team does not describe any anticipated difficulties and how they plan to address them.

Budget is appropriate and justified in relation to the proposed activities.

**Review Type/Type d'évaluation:**	Committee Member 1/Membre de comité 1
**Name of Applicant/Nom du chercheur:**	Andersen, Judith
**Application No./Numéro de demande:**	433650
**Agency/Agence:**	CIHR/IRSC
**Competition/Concours:**	2019-11-05 Team Grant: Mental Wellness in Public Safety Team Grants/Subvention d'équipe : Subventions d'équipe sur le bien-être mental du personnel de la sécurité publique
**Committee/Comité:**	Team Grant: Mental Wellness in Public Safety Team Grants – Full Application/Sub. d'équipe: Sub. d'équipe sur le bien-être mental du personnel de la sécurité publique
**Title/Titre:**	Autonomic Modulation Training: A Biological Approach to Building Resilience and Wellness Capacity Among Police Exposed to Post-Traumatic Stress Injuries (PTSI)

Assessment/Évaluation:

3. Originality of the Proposal

The proposed study is unique in its focus on autonomic nervous system dysregulation instead of a more traditional focus on cognitions, emotions, and behaviors related to PTSI. The proposal articulates a systematic approach to developing and testing the AMT intervention. The use of wearable technology, real-time app-based visualized biofeedback tailored to individuals' unique biorhythms is also novel. The research team is also sensitive to population-specific needs of police officers, as well as issues of mental health stigma in this occupational group. They have also established relationships with several police forces across Canada, which will help increase generalizability of study results. Of note, the proposed study will also provide new insight into the role of sex/gender as autonomic markers of PTSI and resilience, which has been largely ignored in this literature.

4. Applicant(s)

The research team has a long track record of excellence in the proposed area. The team exemplifies a team grant by including experts covering all of the relevant areas for this multidisciplinary project. There is clear complementarity of expertise and synergistic potential to carry out the proposed aims. The team is productive in their research area and have made numerous contributions on AMT and related topics to the scientific literature.

5. Open Science and Knowledge Translation Plan

The researchers outline a detailed knowledge translation, open science, and data management plan that embraces open science principles and practices over the duration of the proposed study. Of note, the research team's relationships with police services should help with recruitment efforts and if found to be efficacious, to more broadly disseminate the intervention to help improve mental health symptoms in the broader community of police officers in Canada.

What is somewhat unclear, however, is *how* the availability of the intervention will be made known to the broader community of police officers. And how can police officers trust the intervention such that their sensitive mental health data will not be shared with others, most notably their employer?

**Review Type/Type d'évaluation:**	Committee Member 1/Membre de comité 1
**Name of Applicant/Nom du chercheur:**	Andersen, Judith
**Application No./Numéro de demande:**	433650
**Agency/Agence:**	CIHR/IRSC
**Competition/Concours:**	2019-11-05 Team Grant: Mental Wellness in Public Safety Team Grants/Subvention d'équipe : Subventions d'équipe sur le bien-être mental du personnel de la sécurité publique
**Committee/Comité:**	Team Grant: Mental Wellness in Public Safety Team Grants – Full Application/Sub. d'équipe: Sub. d'équipe sur le bien-être mental du personnel de la sécurité publique
**Title/Titre:**	Autonomic Modulation Training: A Biological Approach to Building Resilience and Wellness Capacity Among Police Exposed to Post-Traumatic Stress Injuries (PTSI)

Assessment/Évaluation:

One area that could be developed further, however, is regarding the anonymity/confidentiality of enrollment in the intervention program. Will this information ever travel back to an administrator? How might this affect the workers' employment, ability to carry weapons, etc? What if a highly symptomatic police officer is suicidal and requires more in-depth psychiatric care? What procedures are in place to help address these issues?

Review Type/Type d'évaluation: Committee Member 2/Membre de comité 2
Name of Applicant/Nom du chercheur: Andersen, Judith
Application No./Numéro de demande: 433650
Agency/Agence: CIHR/IRSC
Competition/Concours: 2019-11-05 Team Grant: Mental Wellness in Public Safety Team Grants/Subvention d'équipe : Subventions d’équipe sur le bien-être mental du personnel de la sécurité publique
Committee/Comité: Team Grant: Mental Wellness in Public Safety Team Grants – Full Application/Sub. d’équipe: Sub. d’équipe sur le bien-être mental du personnel de la sécurité publique
Title/Titre: Autonomic Modulation Training: A Biological Approach to Building Resilience and Wellness Capacity Among Police Exposed to Post-Traumatic Stress Injuries (PTSI)

Assessment/Évaluation:

1. Impact of Research

Dr. Anderson et al’s research proposal has significant potential to enhance our understanding of sex and gender differences in the body’s objective physiological response to acute and cumulative stress and trauma exposure. Wearable technology to measure real-time biofeedback for first responders will enhance an individual’s self awareness of their ANS reflexes, using a convenient method that is realistic to implement and easy to grasp within stoic workplace cultures. If successfully adopted by test subjects, the indirect impact of this study could lead to significant interest and buy-in from the colleagues of test subjects given the tight-knit culture of many PSP workplaces.

2. Approach

The approach of using HRVB technology to increase self-awareness of the individual stress response represents an exciting new addition to conventional talk therapy approaches to addressing acute and chronic stress reactions. This approach as outlined by Dr. Anderson et al.’s research proposal is a welcome addition to conventional (ie talk therapy) approaches as noted by this quote *“research and clinical practice have shown that directing patients to apply effortful, consciously deployed strategies like reappraisal are not helpful when either subcortical circuitry (e.g., brainstem) or peripheral (e.g., nervous system) physiology is dysregulated.”* Upstream prevention with a focus on health, wellness and resiliency is an important tone to countering mental health stigma in the PSP workplace.

Web-based delivery of an online platform intended for psychoeducational training for police officers requires a high level of motivation and engagement by frontline staff. Buy-in to actively engage and complete this style of training may be a limitation of this study’s approach.

3. Originality of the Proposal

Addressing the physiological underpinnings of the stress response utilizing wearable technology for frontline first responders is a new chapter in assisting police officers in understanding and potentially rewiring ANS reflexes. The proposal also recognizes the most current findings on existing limitations to current resiliency programs for preventing and treating PSP PTSI’s.

4. Track record

Navigating the complexities of a large public safety organization is no easy task and Dr. Anderson’s proven track record as demonstrated in her biography, will enhance buy-in from various levels, most notably, the

Review Type/Type d'évaluation: Committee Member 2/Membre de comité 2
Name of Applicant/Nom du chercheur: Andersen, Judith
Application No./Numéro de demande: 433650
Agency/Agence: CIHR/IRSC
Competition/Concours: 2019-11-05 Team Grant: Mental Wellness in Public Safety Team Grants/Subvention d'équipe : Subventions d’équipe sur le bien-être mental du personnel de la sécurité publique
Committee/Comité: Team Grant: Mental Wellness in Public Safety Team Grants – Full Application/Sub. d’équipe: Sub. d’équipe sur le bien-être mental du personnel de la sécurité publique
Title/Titre: Autonomic Modulation Training: A Biological Approach to Building Resilience and Wellness Capacity Among Police Exposed to Post-Traumatic Stress Injuries (PTSI)

Assessment/Évaluation:

end-user (test subject) of this wearable technology. The widespread buy-in from police departments as reflective through letters of support suggest ease of study implementation.

5. Open Science and Knowledge Translation Plan

“CIHR funds will support the development of modular AMT training that is compatible with learning management systems and can be distributed to PSP sectors across Canada.” This statement indicates open science and also demonstrates how the universal stress response findings are highly applicable across PSP professions.

Overall, the movement beyond a disorder-based approach and towards one that normalizes the acquisition of stress injuries is a novel contribution. This research method and tone is consistent with various provincial (notably BC and Saskatchewan’s) anti-stigma awareness campaigns and will be a significant contribution to open science upon published findings.

**Review Type/Type d'évaluation:**	Committee Member 3/Membre de comité 3
**Name of Applicant/Nom du chercheur:**	Andersen, Judith
**Application No./Numéro de demande:**	433650
**Agency/Agence:**	CIHR/IRSC
**Competition/Concours:**	2019-11-05 Team Grant: Mental Wellness in Public Safety Team Grants/Subvention d'équipe : Subventions d'équipe sur le bien-être mental du personnel de la sécurité publique
**Committee/Comité:**	Team Grant: Mental Wellness in Public Safety Team Grants – Full Application/Sub. d'équipe: Sub. d'équipe sur le bien-être mental du personnel de la sécurité publique
**Title/Titre:**	Autonomic Modulation Training: A Biological Approach to Building Resilience and Wellness Capacity Among Police Exposed to Post-Traumatic Stress Injuries (PTSI)

Assessment/Évaluation:

Criterion #1: Research Approach

This application was well laid out, easy to understand, with clear goals and objectives. However, it was not apparent, the intended application of the online AMT post-study. Included in the application was an educational component that physical and psychological factors do not happen independently of each other. There was a good explanation of the psychophysiological process of how the Sympathetic Nervous System (SNS) and Parasympathetic Nervous System (PNS) are interconnected to the physical body. The visual aids and the figures that included explanations of the various measurements and questionnaires were very helpful. This proposal took great care in explaining Heart Rate Variability Biofeedback (HRVB), and why it is needed and how it is applied to this research. The bottom up approach was refreshing, however, there is concern that it is only individual focused.

Careful consideration was given to knowledge transfer including publications, partnerships, face to face meetings, although it appears there is room to reach a broader audience, such as, conference presentations etc.

Research supports that AMT in clinical and non-clinical setting are effective, hence, I am a little confused for the need of this study to be so robust. It is understood that it specifically addresses police officers and it will address the sex and gender to baseline biological differences in PTSI symptoms and the effectiveness of AMT intervention, however, I am unsure if the requested funding amount is fiscally responsible with the anticipated outcome(s).

In addition to the AMT, the proposal also intends to integrate psychoeducational modules focused on developing meta-cognitive skills. In addition, participants will receive further training on effective coping skills and emotional regulation. I do not understand how it will be separated in the post–measure analysis, as to whether the AMT solely affected the outcome of lowering PTSI symptoms and increased their resilience and wellness capacity or if these other additional online modules and training also had an affect on the post measurements. The proposal states that the *"reduction of PTSI symptoms and improvement in resilience are uniquely accounted for by the AMT intervention"*, but what does this mean? For example, is it taken into account additional "treatments" (medicinal or non-medicinal) while participating in the study?

The proposal indicates that self regulation by conditioning the HRV and RSA with the proposed online AMT has short-term and long-term benefits, however, the post measurements stops after the 6 weeks of implementing the AMT. This would only indicate the immediate outcome and will not measure any long-term benefits as indicated. Are the benefits of AMT only short-term while being

Review Type/Type d'évaluation: Committee Member 3/Membre de comité 3
Name of Applicant/Nom du chercheur: Andersen, Judith
Application No./Numéro de demande: 433650
Agency/Agence: CIHR/IRSC
Competition/Concours: 2019-11-05 Team Grant: Mental Wellness in Public Safety Team Grants/Subvention d'équipe : Subventions d’équipe sur le bien-être mental du personnel de la sécurité publique
Committee/Comité: Team Grant: Mental Wellness in Public Safety Team Grants – Full Application/Sub. d’équipe: Sub. d’équipe sur le bien-être mental du personnel de la sécurité publique
Title/Titre: Autonomic Modulation Training: A Biological Approach to Building Resilience and Wellness Capacity Among Police Exposed to Post-Traumatic Stress Injuries (PTSI)

Assessment/Évaluation:

used daily or do the benefits continue long after daily use of the online AMT is discontinued?

Criterion #2: Originality of the Proposal
It was difficult to understand why a wed-based designed and delivery of AMT is required when there are already mobile apps and other easily accessible HRVB widely available. Is this a duplication of efforts?

Having said that, having access to an AMT easily accessible (especially for rural and remote PSPs), having autonomy and easy to use would be welcomed, but again, in my experience, it would not be utilized as much as one would hope in the “real world”. Understanding why this is, how to improve ‘buy-in” and understanding the challenges would be important, therefore, digging deeper into engagement and adherence would be recommended.

Highlighted was a knowledge gap in current research of how sex and gender may affect receptivity and treatment effects of HRVB. It is also worthy to note the originality looking in depth the organizational and occupational stresses taken into consideration, specifically for female officers. It is also welcomed to see the flexibility and effort put into the analysis to prominent emerging themes.

Criterion #3: Applicant(s)
There appears to be a concerted effort to form a multi-disciplinary team with a wide range of expertise and some activities that garnished user-informed insights. Although it wasn’t immediately apparent, it was found in the budget proposal there is intentions to utilize Postdoctoral fellows, graduate and summer students.

As a knowledge-user peer reviewer, it is difficult to comment beyond the above observations.

Criterion #4: Environment for the Research
I think there is a good plan laid out to advertise for recruitment, however, I am concerned of the small margin that is accounted for with attrition. Given the workload demand, unpredictable nature of the work, shift work, I think it will be a challenge recruiting police officers that can commit to the study and maintaining high attrition. For example, committing to 1 hour each week at the same time each time, will be difficult, and this is only one of the many commitments required for the study. I think this attrition is going to be more of an issue than is accounted for in the study. Having said that, there is a small financial incentive that may assist with follow through.

I do like the forethought into those PSPs that self-assess as severe and on the high end of the cut off

Review Type/Type d'évaluation: Committee Member 3/Membre de comité 3
Name of Applicant/Nom du chercheur: Andersen, Judith
Application No./Numéro de demande: 433650
Agency/Agence: CIHR/IRSC
Competition/Concours: 2019-11-05 Team Grant: Mental Wellness in Public Safety Team Grants/Subvention d'équipe : Subventions d’équipe sur le bien-être mental du personnel de la sécurité publique
Committee/Comité: Team Grant: Mental Wellness in Public Safety Team Grants – Full Application/Sub. d’équipe: Sub. d’équipe sur le bien-être mental du personnel de la sécurité publique
Title/Titre: Autonomic Modulation Training: A Biological Approach to Building Resilience and Wellness Capacity Among Police Exposed to Post-Traumatic Stress Injuries (PTSI)

Assessment/Évaluation:

scores have a follow up plan to provide additional support to the PSP. It is confusing though, that this PSP would still have the option to participate in the study, yet they are receiving additional supports outside of the cope of the study. Is this taken into consideration with the results? It is also not clear if this would affect their ability to continue to continue their duties as a police officer, once this is known. Who will have access to this information? Does this information go back to the organization? What are the ethical obligations if this occurs?

Data management, exchange and security was considered in this proposal, including leveraging already existing platforms and systems, which is fiscally responsible and helps to mitigate margins of error.

Criterion #5: Impact of the Research
Although the proposal is well written and laid out, I am not certain that this research will have as a high impact. There are several reasons which are as follows:

1. In the context of building resilience in PSPs, as are most of the research to date, this study is individual-focused and does little to consider the complexity of multiple factors that play a significant role in PTSI resilience, health and wellness capacity. As such, it is suggested that this will have at a micro-level, as opposed to a macro-level.

2. As a knowledge-user having utilized an HRVB myself, it was an issue in continuing to utilize the device while at home/work and away from the clinical setting where time was set aside, and a clinician was present. It is recognized that there are focus groups to include qualitative data, however, outside of the study and in the "real world", there are no financial incentives to do HRVB and that is a realistic concern. Although the AMT may available to the Canadian PSP, will it be utilized? In short, if it is not being used, how can it be effective? Understanding the challenges surrounding its continued use would be very valuable.

3. It was not clear in the proposal if the AMT is intended to be for PSPs with low, moderate or severe symptoms or combination. As a knowledge-user, it is believed that prevention and early intervention is the key to faster and a successful recovery, however, very few PSPs proactively build their resilience until there is a need for it. I pose the question: realistically how many PSPs on the low end of severity would seek this out and adhere to utilizing this daily, as a prevention with increasing their well-being and resilience?

4. In my experience, many PSPs will not come forward, even if they suspect the onset of MH issues and /or PTSD symptoms, for several reasons, including but not limited to; stigma, myths about PTSD, can be

Review Type/Type d'évaluation: Committee Member 3/Membre de comité 3
Name of Applicant/Nom du chercheur: Andersen, Judith
Application No./Numéro de demande: 433650
Agency/Agence: CIHR/IRSC
Competition/Concours: 2019-11-05 Team Grant: Mental Wellness in Public Safety Team Grants/Subvention d'équipe : Subventions d'équipe sur le bien-être mental du personnel de la sécurité publique
Committee/Comité: Team Grant: Mental Wellness in Public Safety Team Grants – Full Application/Sub. d'équipe: Sub. d'équipe sur le bien-être mental du personnel de la sécurité publique
Title/Titre: Autonomic Modulation Training: A Biological Approach to Building Resilience and Wellness Capacity Among Police Exposed to Post-Traumatic Stress Injuries (PTSI)

Assessment/Évaluation:

career ending and/or limiting, possible machoism. Their PTSD PSPs may not disclose until there is such a significant deviation of baseline behavior that draws attention to themselves, thus, surmising that they are on the moderate-severe spectrum of PTSD and its related symptomatology. Again, I pose the question: given the heightened state of the PSP with **symptomatology, how many would embark in an AMT on their own and what are the challenges associated with it?**

5. It is also concerning the application after the study may be too expensive for PSP to utilize. Will PSPs be able to afford the hardware needed (ie/ Fitbit, Apple iWatch) and the software costs , if it is not covered with health benefits? This can be very costly.

Summary:
This application does a great job of explaining the psychophysiological and the biomechanics of HRV. Previous research has indicated that physiological functioning has shown success in building resilience and wellness capacity, however, there is concern of how realistic this would be in the "real world" with PSP engagement and adherence to an online AMT. This is not to say that the AMT would not be beneficial, however, it would only be beneficial if it is being utilized. Additionally, it is concerning that this study may have some duplication, it is individual-focused and is not expected to have high impact.

Review Type/Type d'évaluation: SO Notes /Notes de l'agent scientifique
Name of Applicant/Nom du chercheur: Andersen, Judith
Application No./Numéro de demande: 433650
Agency/Agence: CIHR/IRSC
Competition/Concours: 2019-11-05 Team Grant: Mental Wellness in Public Safety Team Grants/Subvention d'équipe : Subventions d’équipe sur le bien-être mental du personnel de la sécurité publique
Committee/Comité: Team Grant: Mental Wellness in Public Safety Team Grants – Full Application/Sub. d’équipe: Sub. d’équipe sur le bien-être mental du personnel de la sécurité publique
Title/Titre: Autonomic Modulation Training: A Biological Approach to Building Resilience and Wellness Capacity Among Police Exposed to Post-Traumatic Stress Injuries (PTSI)

Assessment/Évaluation:

The committee felt that this grant was well-written and well justified and commented on the innovative bottom-up focus. It has the potential to develop a scalable intervention likely to be adopted. The value of the sex and gender lens for the proposed work was also noted. Other strengths include the fit of this proposal to the move in the field to go beyond talk therapy – that is, the growing receptivity and buy-in of this kind of approach -- as well as the potential to destigmatize PTSI. The track record of the team and the letters of support from a wide number of stakeholders were also considered plusses.

A major committee concern was the pre-post design which lacked a control or even a treatment-as-usual condition and therefore seriously compromised the study’s ability to arrive at robust findings. There was also discussion around whether the intervention would actually be adopted and how long-term benefits would be assessed given the study’s described timeline. Whether the web-based application would actually be used was raised, but the point that this might differ depending on the age of the participant was also discussed. How attrition (expected given the typical police workload) and possible concurrent treatment would be addressed in the analyses and therefore the interpretation and conclusions were also raised.

Addressing these committee concerns would strengthen this application.

Budget concerns: none were raised.
